# Transcriptomic analyses reveal proinflammatory activation of human brain microvascular endothelial cells by aging-associated peptide medin and reversal by nanoliposomes

**DOI:** 10.1038/s41598-023-45959-7

**Published:** 2023-11-01

**Authors:** Yining Zhang, Nina Karamanova, Kaleb T. Morrow, Jillian Madine, Seth Truran, Maria Lozoya, Volkmar Weissig, Ming Li, Mehdi Nikkhah, Jin G. Park, Raymond Q. Migrino

**Affiliations:** 1 Center for Personalized Diagnostics, Biodesign Institute, Arizona State University, Tempe, AZ USA; 2 Phoenix Veterans Affairs Healthcare System, 650 E. Indian School Road, Phoenix, AZ 85022 USA; 3 University of Liverpool, Liverpool, UK; 4 Midwestern University, Glendale, AZ USA; 5 University of Arizona College of Medicine-Phoenix, Phoenix, AZ USA; 6 School of Biological and Health Systems Engineering, Arizona State University, Tempe, USA

**Keywords:** Cerebrovascular disorders, Alzheimer's disease, Vascular diseases, Ageing

## Abstract

Medin is a common vascular amyloidogenic peptide recently implicated in Alzheimer’s disease (AD) and vascular dementia and its pathology remains unknown. We aim to identify changes in transcriptomic profiles and pathways in human brain microvascular endothelial cells (HBMVECs) exposed to medin, compare that to exposure to β-amyloid (Aβ) and evaluate protection by monosialoganglioside-containing nanoliposomes (NL). HBMVECs were exposed for 20 h to medin (5 µM) without or with Aβ(1-42) (2 µM) or NL (300 µg/mL), and RNA-seq with signaling pathway analyses were performed. Separately, reverse transcription polymerase chain reaction of select identified genes was done in HBMVECs treated with medin (5 µM) without or with NFκB inhibitor RO106-9920 (10 µM) or NL (300 µg/mL). Medin caused upregulation of pro-inflammatory genes that was not aggravated by Aβ42 co-treatment but reversed by NL. Pathway analysis on differentially expressed genes revealed multiple pro-inflammatory signaling pathways, such as the tumor necrosis factor (TNF) and the nuclear factor-κB (NFkB) signaling pathways, were affected specifically by medin treatment. RO106-9920 and NL reduced medin-induced pro-inflammatory activation. Medin induced endothelial cell pro-inflammatory signaling in part via NFκB that was reversed by NL. This could have potential implications in the pathogenesis and treatment of vascular aging, AD and vascular dementia.

## Introduction

Medin is a 50-amino acid peptide formed from the cleavage of parent protein milk fat globule-EGF factor 8 (MFGE8) and is the peptide component of one of the most common human amyloidoses^1,2^. Medin accumulates in multiple arterial beds with aging, including the cerebral arteries and brain parenchymal arteries^3,4^. Cerebral arterial medin burden is associated with Alzheimer’s disease (AD) and vascular dementia in elderly brain donors and levels correlate with plaque density score, neurofibrillary tangle and cerebral white matter lesions^3^. Preclinical investigation shows that genetic deletion of medin-containing C2 domain of Mfge8 resulted in preservation of cerebrovascular function in aging C57BL/6 mice^5^. Recently it was shown in several transgenic AD mouse models that genetic deletion of the C2 domain of Mfge8 resulted in reduced cerebral amyloid angiopathy and that medin enhances the aggregation of β-amyloid (Aβ) while colocalizing with Aβ plaques^6^. These findings suggest a prominent role of medin in mediating or modulating vascular aging and pathologies resulting from it^6^. The mechanisms underlying medin pathology remain poorly understood. In human cerebral arterial tissue ex vivo and cellular in vitro experiments, physiologic concentrations of recombinant medin resulted in endothelial and smooth muscle dysfunction induced by oxidative and nitrative stress^7,8^. Medin was also found to induce endothelial pro-inflammatory activation that is mediated, at least in part, through the receptor for advanced glycation end-products and nuclear factor kappa B (NFκB)^8,9^ and that this could trigger astrocyte pro-inflammatory activation in paracrine fashion^9^. Among brain cell types, endothelial cells and astrocytes showed the greatest *Mfge8* gene expression^10^ and endothelial dysfunction was shown to be one of medin’s pathophysiologic effects^8,9^. Somatosensory cortex vasodilation following hindlimb stimulation, an endothelium-dependent neurovascular coupling response^11^ that is impaired with aging, improved in aged C57BL/6 mice with genetic deletion of medin-containing C2 domain of *Mfge8* compared to wild type controls^5^. These suggest the importance of studying medin pathophysiology on endothelial cells.

Nanoliposomes are artificial phospholipid vesicles < 100 nm in size considered to be of therapeutic value because they are nonimmunogenic, fully biodegradable, structurally versatile and with good safety profile^12^. We developed and showed that monosialoganglioside-containing nanoliposomes (NL, composed of 70% phosphatidylcholine, 25% cholesterol and 5% monosialoganglioside) reversed amyloid light chain- and medin-induced endothelial dysfunction and medin-induced endothelial cell pro-inflammatory activation in part by triggering nuclear factor erythroid 2-related factor 2 (Nrf2)-dependent antioxidant stress response and preventing NFκB activation^4,9^. To gain insights on molecular basis of pathological action of medin on endothelial dysfunction, we performed an unbiased RNA-Seq-based transcriptomic profiling on human brain microvascular endothelial cells (HBMVECs) treated with various combinations of medin, Aβ(1-42), and NL. Differential expression and pathway enrichment analyses were then performed between the treatment conditions, and the key identified genes were validated by RT-PCR.

## Results

### Global transcriptional changes induced by medin, Aβ, and NL in HBMVECs

To investigate molecular impact of medin on HBMVECs, three independent sets of seven treatment groups of HBMVECs were prepared and analyzed by RNA-seq, which included cells treated for 20 h with either Vehicle (culture media), Medin (5 µM, a physiologic concentration found in human tissues^4,8^), Scrambled Medin (scrMedin, 5 µM), Aβ(1-42) (2 µM, biologically relevant dose that induced endothelial dysfunction in human tissue^13^), NL (300 µg/mL, dose that reversed medin-induced endothelial dysfunction^4^) and co-treatment groups of Medin + Aβ and Medin + NL. After batch effect correction, Principal Component Analysis (PCA) identified an outlier within the scrMedin group, which was excluded from further analysis (Supplement Fig. 1). The gene expression results of each treatment are shown in Supplement Table 1.

**Figure 1:**
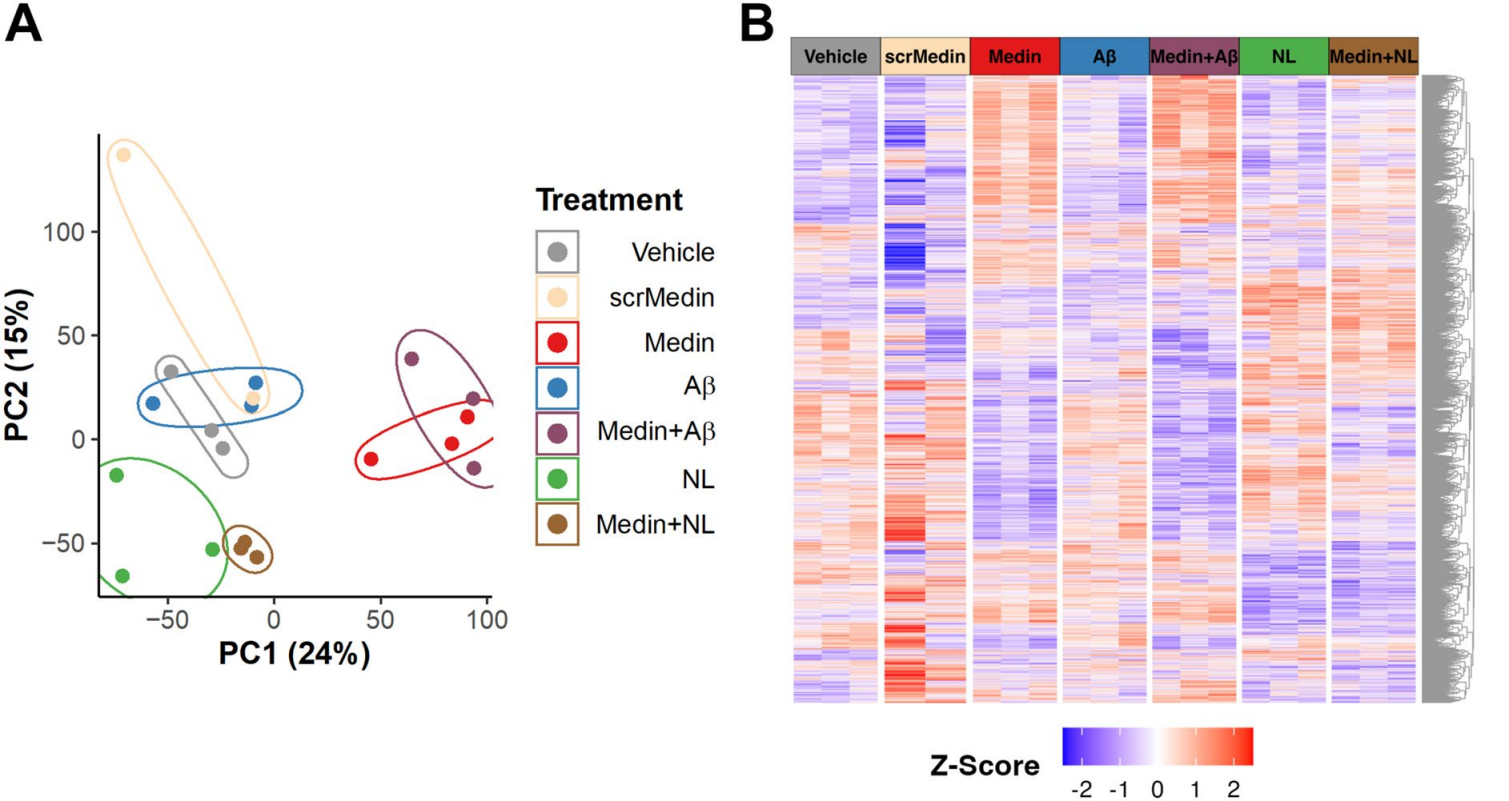
Transcriptomic Analyses. Differences in global transcriptomics profiles of medin-, Aβ-, and NL-treated HBMVECs. (**A**) PCA was applied to gene expression data on 7 different treatment groups, each in triplicates. Prior to the analysis, the batch effect was corrected, and one outlier sample in the scrMedin group was removed. The x-axis represents the first principal component, which accounts for 24% of total variance in the data, while the y-axis corresponds to the second principal component, explaining 15% of the variance. Samples are colored based on their respective treatment. (**B**) The heat map shows row- or gene-wise Z score transformed gene expression levels of 7704 DEGs identified by pairwise linear model analysis. Each row represents a gene, while each column corresponds to an individual sample. The columns are sorted by treatments, while the rows are clustered based on the Euclidean distances.

When global transcriptomes of individual samples were compared by PCA (Fig. 1A), an unsupervised dimensional reduction method, samples in each experimental group were closely clustered, indicating that similar gene expression profiles were shared among the biological replicates, largely distinct from other groups. Most notably, whereas the negative control groups of Vehicle and scrMedin were closely positioned as expected, Aβ-treated cells were virtually overlapped with the vehicle-treated cells, implying that Aβ exerted little impact on gene expression in HBMVECs. Likewise, the co-treatment group of Medin + Aβ was also positioned adjacent to the Medin-treated group, further indicating minimal cellular effects of Aβ.

To identify differentially expressed genes (DEGs) over the Vehicle group across different treatment groups, pairwise linear model-based analyses were then performed with thresholds of multiple-testing-adjusted *p* (or False Discovery Rate, FDR) < 0.05 and absolute fold changes over 2 (Supplement Table 2). In agreement with the PCA results, the heat map (Fig. 1B) of a total of 7704 identified DEGs across the groups showed that Aβ and Medin + Aβ groups had very similar gene expression profiles to those of Vehicle and Medin groups, respectively, whereas Medin, Medin + NL, and NL groups had distinctive profiles.

### Transcriptional changes in HBMVECs induced by medin

**Figure 2:**
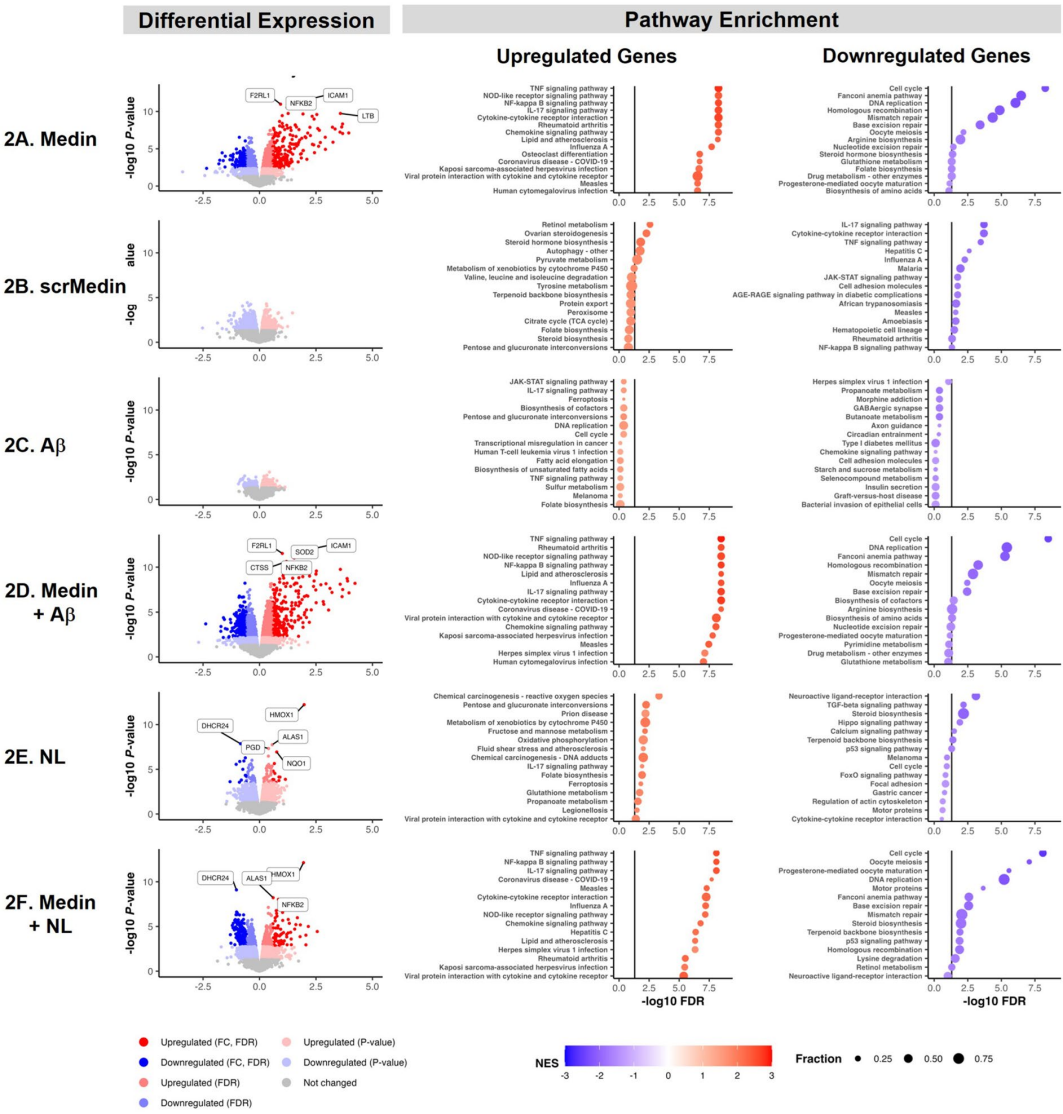
Differential Gene Expression and Pathway Enrichment Analyses. Left panels show volcano plots of differential expression statistics versus vehicle-treated group in the medin-treated HBMVECs (**A**), scrambled medin-treated group (**B**), Aβ-treated group (**C**), medin + Aβ-treated group (**C**), NL-treated group (**E**) and medin + NL-treated group (**F**) (n = 3 each treatment). The x-axis represents the log2 fold change in expression in the Medin group over the Vehicle group. The DEGs are colored according to their log2 fold change (FC), p-value, and False Discovery Rate (FDR). Genes with p-values bigger than 0.05 are colored black and are considered unchanged. Genes with a p-value less than 0.05 and a log2 FC greater than 0 are considered upregulated genes and colored red, while genes with a p-value less than 0.05 and a log2 FC less than 0 are considered downregulated genes and colored blue. According to significance, upregulated or downregulated genes are colored with different shades: genes with log2 FC greater than 1.5 or less than 1.5 and FDR less than 0.05 are the darkest, followed by genes with FDR less than 0.05 but log2 FC between 1.5 and 1.5, and the lightest color is assigned to genes with FDR greater than 0.05. Middle panel shows top enriched upregulated gene pathways and right panel shows top enriched downregulated gene pathways identified by the Gene Set Enrichment Analysis (GSEA). The size of the dot reflects the fraction of DEGs in each pathway. The dot color indicates the Normalized Enrichment Score (NES).

**Figure 3:**
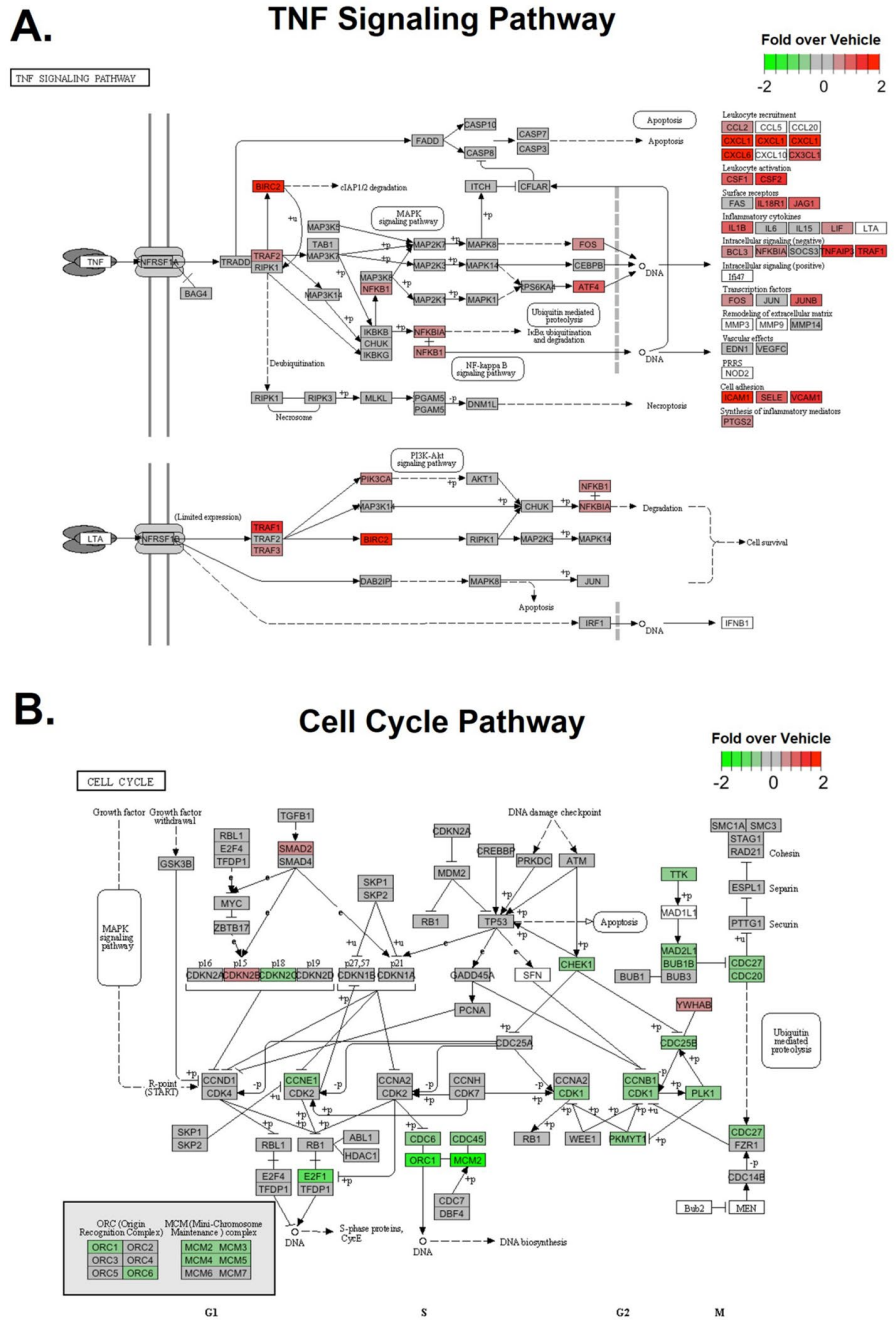
Gene expression profiles of top KEGG pathways, TNF signaling pathway and cell cycle pathway, enriched in upregulated (**A**) and downregulated (**B**) genes, respectively, by medin treatment in HBMVECs.

As the first between-group comparison, we examined how the transcriptomic profile of HBMVECs was altered following exposure medin versus the vehicle-treated controls, and the DEG analysis identified 678 upregulated and 542 downregulated genes (FDR < 0.05, Fig. 2A). Gene enrichment analyses showed that genes associated with inflammatory signaling were the most upregulated, including genes involved in tumor necrosis factor (TNF) signaling pathway (Fig. 3A, Supplement Table 3), nucleotide-binding and oligomerization domain (NOD)-like receptor signaling, NFκB signaling and interleukin (IL)-17 signaling pathways. Significantly downregulated genes were involved in cell cycle pathway, Fanconi anemia pathways, and DNA replication (Figs. 2A and 3B). In contrast, the scrambled medin did not induce significant changes in gene expression, i.e., no differentially expressed genes have FDR < 0.05 (Fig. 2B), suggesting that these effects were medin-specific.

### Transcriptional changes in HBMVECs induced by Aβ(1-42) and medin + Aβ(1-42)

Aβ(1-42) is a peptide fragment cleaved from parent protein amyloid precursor protein and is a major peptide component of amyloid plaques and cerebral amyloid angiopathy in AD^14–16^. Amyloid found in plaques and cerebral amyloid angiopathy in AD mouse models was recently shown to contain both medin and Aβ peptides^6^. Compared to medin, exposure of HBMVECs to physiologic dose of Aβ(1-42) did not induce significant gene expression changes (Fig. 2C, Supplement Table 2). In addition, exposure of HBMVECs to both medin and Aβ(1-42) led to upregulation of genes related to proinflammatory signaling of similar magnitude to effects seen with medin alone (Fig. 2D, Supplement Table 3), suggesting that endothelial pro-inflammatory activation is driven mainly by medin with no additive or synergistic effect by Aβ(1-42).

### Protective effect of NL against medin-induced changes in HBMVECs

We previously showed that NL prevented medin-induced endothelial cytotoxicity likely through Nrf2-dependent upregulation of gene and protein expressions of antioxidant enzymes heme oxygenase-1 (HO1), NAD(P)H quinone oxidoreductase 1 (NQO1) and superoxide dismutase1 (SOD1), while also preventing medin-induced NFκB-mediated proinflammatory activation^4^. In agreement, we report that co-treatment of NL with medin attenuated the upregulation of pro-inflammatory genes by medin (Fig. 2F, Supplement Table 2). NL alone resulted in upregulation of a small set of genes (Fig. 2E) including heme oxygenase 1 (*HMOX1*), consistent with our prior findings, as well as *ALAS1,* which encodes the mitochondrial enzyme that catalyzes the rate-limiting step in heme (iron-protoporphyrin) biosynthesis. Genes upregulated by NLs were associated with chemical carcinogenesis-reactive oxygen species, while genes downregulated by NLs were associated with neuroactive ligand-receptor interaction and transforming growth factor-beta (TGF-β) signaling (Supplement Table 3).

**Figure 4:**
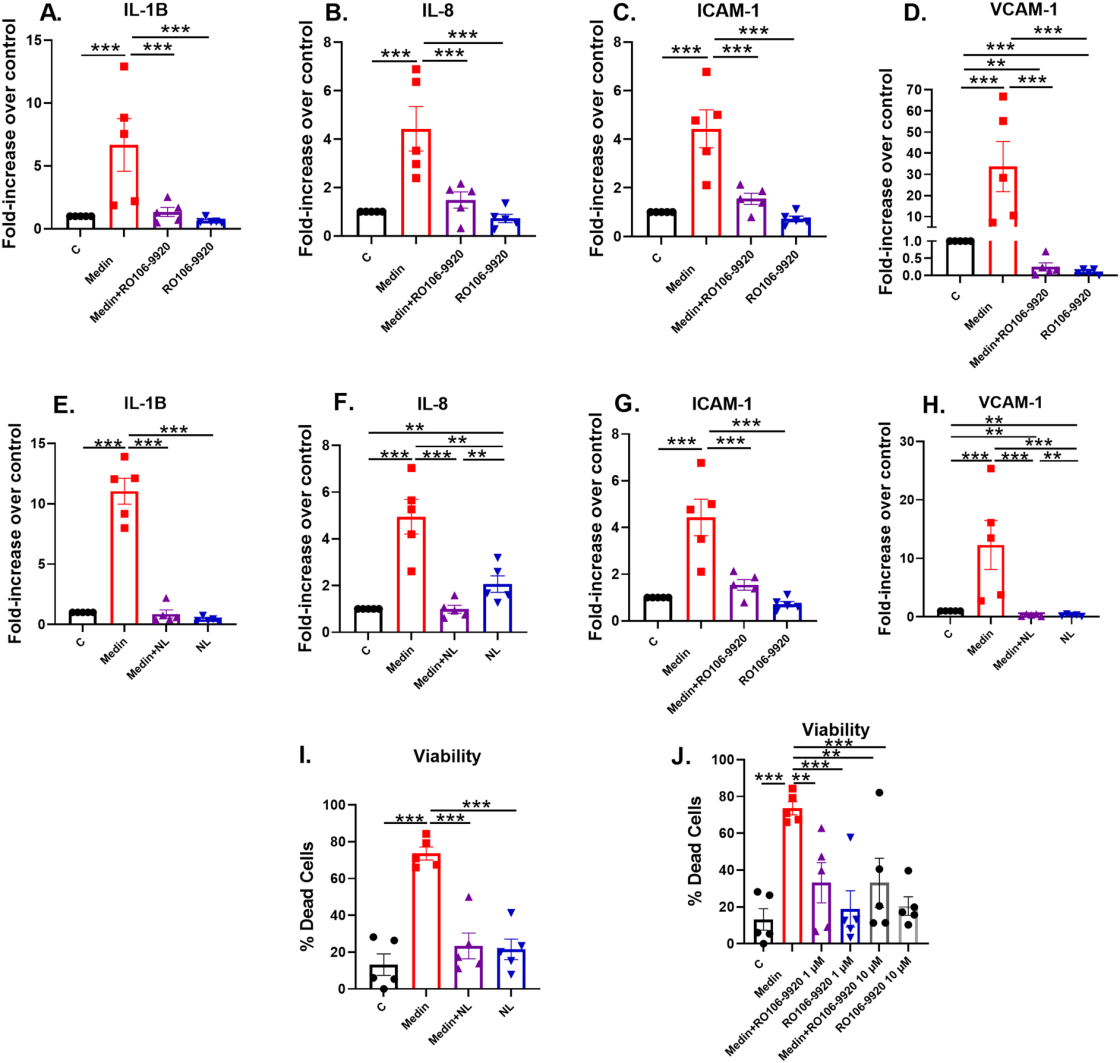
(**A**–**D**) rt-PCR of HBMVECs treated for 20 h with medin and NFκB inhibitor RO106-9920 (N = 5 each). There was significant elevation in gene expressions of IL-1B, IL-8, ICAM-1 and VCAM-1 following 20 h of treatment with medin 5 µM. Co-treatment with RO106-9920 (10 µM) abolished or attenuated the increased cytokine/chemokine gene expressions. (**E**–**H**) Medin (5 µM) increased the gene expressions of the cytokines and chemokines and this increase was attenuated by co-treatment with NL (N = 5 each). (**I**) Medin (5 µM) increased HBMVEC cell death after 20 h exposure versus vehicle control that was reversed by NL. (**J**) Similarly, co-treatment of medin with RO106-9920 (either 1 or 10 µM) reduced cell death versus medin treatment alone. There was no significant difference in cell viability between vehicle control and RO106-9920 treated cells (N = 5 each). One-way repeated measures analyses of variance were performed followed by pairwise multiple comparison methods using Holm-Sidak method. ***p < 0.001, **p < 0.01 represent unadjusted p values that are below critical levels required by the multiple comparison procedure.

In separate experiments, HBMVECs treated with medin for 20 h showed increased cell death (reduced viability) that was reversed by co-treatment with NL. Similarly, co-treatment with RO106-9920 at both 1 and 10 µM doses showed reduced cell death versus medin treatment alone (Fig. 4I–J). NL and RO106-9920 (1 and 10 µM) treatments did not show significantly higher cell death than vehicle control (Fig. 4I–J).

### RT-PCR gene expression analyses

As RNA-seq showed upregulation of inflammatory genes linked with NFκB signaling, we performed RT-PCR gene expression assays, focusing on the cytokine/chemokine genes downstream of NFκB signaling that were upregulated in RNA-seq analyses. In separate experiments, HBMVECs were exposed to medin without and with RO106-9920, a small molecule specific inhibitor of NFκB^17^. Similar to results from RNA-seq, RT-PCR showed significantly increased gene expressions of *IL1B*, *IL8*, intercellular adhesion molecule 1 (*ICAM1*) and vascular cell adhesion molecule 1 (*VCAM1*) following 20 h of exposure to medin compared to vehicle control (Fig. 4A–D). Co-treatment with RO106-9920 attenuated or prevented the increases. These confirm RNA-seq data showing NFκB-mediated pro-inflammatory activation by medin in HBMVECs.

We previously showed that NL protected against medin-induced human umbilical vein endothelial cell pro-inflammatory activation. In this work, we treated HBMVECs with medin without or with NL and found that NL attenuated the increased gene expression of *IL1B*, *IL8*, *ICAM1* and *VCAM1* (Fig. 4E–H), consistent with our RNA-seq-based findings.

## Discussion

Our results show that 20-h exposure to physiologic dose of recombinant medin in HBMVECs elicited an array of transcriptomic changes that predominantly involved upregulation of genes involved in inflammatory signaling pathways, with genes involved in TNF signaling, NOD-like receptor signaling, NFκB signaling and IL-17 signaling being the top hits. These signaling pathways share in common upregulation of NFκB activation-dependent proinflammatory cytokines/chemokines such as IL-1B, CXCL-8/IL-8, ICAM-1 and VCAM-1. RT-PCR confirmed the upregulated gene expressions of these NFκB-induced cytokines/chemokines. To confirm the increased gene expression of cytokines/chemokines observed in the RNAseq profile, we performed RT-PCR that also showed medin increasing IL-1B, IL-8, ICAM-1 and VCAM-1. RO106-9920 is a small molecule inhibitor of NFκB activation through ubiquitination of IκBαee^17^. Co-treatment of medin with RO106-9920 reduced gene expression of these cytokines/chemokines versus medin alone, confirming that medin’s pro-inflammatory effect is via NFκB activation. These results are consistent with our prior limited observation of pro-inflammatory activation of human umbilical vein endothelial cells by medin that was mediated via NFκB pathway^8^. The results have implications in advancing our understanding of the mechanisms of vascular aging and the myriad of pathologies that arise from it. Medin is the most common human amyloid^1^ and in a routine autopsy of 18 individuals 57 years old and higher, medin amyloid deposition was found in the aortas of all patients and in the basilar artery in 8 of 17 subjects^2^. Medin amyloid accumulates in the vasculature with aging with aortic medin present in 86% of subjects over 55 years old versus 20% in younger patients^8^. Medin was also shown to accumulate in the brain parenchymal arteries in elderly brain donor subjects, and among cerebrovascular pathologies, cerebral arterial medin burden had the strongest association with AD and vascular dementia^3^. Medin is therefore proposed to be one of the key novel mediators of vascular aging and vascular aging-related pathologies. Chronic indolent vascular inflammation is one of the hallmarks of vascular aging phenotype, independent of, although exacerbated by, presence of traditional cardiovascular metabolic risk factors^18^. In this study we showed that medin induces profound pro-inflammatory activation of HBMVECs. The implications go beyond effects on vascular inflammation as medin-induced endothelial proinflammatory activation could induce astrocyte activation and neuroinflammation through paracrine effects^4^.

Genes involved in the cell cycle signaling and the DNA replication pathways were the most downregulated by medin. These pathways may have physiologic relevance as prior work showed that medin reduced endothelial cell proliferation; endothelial cell viability was also reduced through oxidative and nitrative stress, with viability restored when co-treated with peroxynitrite catalyst^8^. The genes upregulated and downregulated by medin, including the signaling pathways affected, were either not affected or affected several log-fold less by scrambled medin, suggesting that medin’s effects are specific to the peptide.

Aβ(1-42) is one of two common alloforms of Aβ found in amyloid deposits in AD; it forms fibrils and has more enhanced neurotoxicity compared to the other common alloform Aβ(1-40)^19^. Both Aβ(1-42) and medin induce endothelial dysfunction in human arterioles^8,13,20,21^ and amyloid deposits in cerebral arteries of AD brain donors^3^ and AD transgenic mouse models^6^ both contain Aβ and medin. Indeed, medin was found to enhance Aβ aggregation and genetic deletion of medin from parent protein Mfge8 reduced cerebral amyloid angiopathy in AD mouse models^6^. Therefore, it was of interest to compare the effects of Aβ(1-42) and medin in HBMVECs as well as determine the effects of the combination. In contrast to medin, no signaling pathway genes were significantly upregulated or downregulated in HBMVECs with exposure to Aβ(1-42). When medin and Aβ(1-42) were co-treated, the gene and signaling pathway profiles were similar in pattern and magnitude to those of medin alone. This suggests that the pro-inflammatory transcriptomic change is driven mainly by medin, and that Aβ(1-42) does not have any additive, synergistic or reductive effect when co-treated with medin. A similar pattern was also observed for the downregulated genes enriched with cell cycle and DNA replication signaling pathway between medin and medin + Aβ treatments. A potential implication of these findings is that medin may be the more relevant treatment target when compared to Aβ(1-42) in terms of preventing or reversing endothelial pro-inflammatory activation and cell survival. Future testing should confirm whether other Aβ alloforms will show similar or variant results.

NLs are < 100 µm nanoparticles composed of phospholipids. We previously showed that NLs protected against Aβ-induced^13,21^ and medin-induced^4^ endothelial dysfunction and restored endothelial cell viability. Monosialoganglioside-containing NLs used in this study were previously found to protect against medin-induced cell toxicity through a Nrf2-dependent upregulation of gene and protein expression of antioxidant enzymes HO-1, NQO1 and SOD11^4^. Similarly, NL also attenuated medin-induced pro-inflammatory activation of HUVECs by preventing NFκB activation^4^. In the current study, NL caused significant upregulation of HO-1 and NQO1, genes involved in chemical carcinogenesis-reactive oxygen species signaling pathway, consistent with our previous findings. Genes involved with neuroactive ligand-receptor interaction signaling. TGF-β signaling and steroid biosynthesis were the most significantly downregulated by NLs. Co-treatment of medin with NL on HBMVEC attenuated upregulation of pro-inflammatory genes and signaling pathways consistent with our prior findings^4^. This was confirmed with RT-PCR wherein gene expressions of *IL1B*, *IL8*, *ICAM1* and *VCAM1* were reduced when medin was co-treated with NLs versus medin alone. Of interest, despite prior report of NL rescuing cell viability of medin-treated HUVECs, NL + medin did not change the downregulation of genes associated with cell cycle signaling when compared to medin treatment alone. Overall, the results show the protective effect of NL in medin-induced endothelial pro-inflammatory activation and confirm NL’s effects in upregulation of antioxidant genes. This demonstrates the potential of NL as a novel therapeutic agent against medin vascular toxicity.

The study is limited in describing only differential mRNA expressions which may not be necessarily translated to the protein level changes. However, we previously showed that medin increased HUVEC IL-6, IL-8, ICAM-1 and VCAM-1 gene and protein expressions^4,8^. We used single doses of medin and Aβ(1-42) based on concentrations found in human tissues as well doses confirmed to cause endothelial dysfunction in human arterioles, and the effects of lower or higher doses, especially as regards medin and Aβ interactions, are not known and warrant further studies. Although the sample size per treatment for RNA-seq analyses is relatively small (N = 3 biologic replicates), the profound and significant gene expression changes induced by medin especially those related to inflammatory signaling, which were confirmed by rt-PCR, highlight the validity of our findings. However, at the same time, other signaling pathways could be revealed with larger sample size. Although both medin and Aβ42 were added in non-aggregated/soluble forms, differential aggregation kinetics between the two peptides during treatment period could have affected the outcomes and should be studied in the future. Similar preparations of medin and Aβ42 were given for 1 h to ex vivo human adipose and leptomeningeal arterioles in our prior works^4,7,13,22^, and both induced endothelial dysfunction that was reversed by nanoliposomes, which when viewed in relation to our current results suggest shared and variant mechanisms of injury between the two amyloidogenic peptides that could be relevant in understanding the pathophysiology and treatment of AD, vascular dementia and other aging-related vascular diseases. The nature of medin cleavage from MFGE8 and its vascular accumulation remain poorly understood; future studies should investigate signaling pathways and enzymatic mediators that lead to MFGE8 cleavage, focusing on cellular processing/homeostasis that maintain balance in controlling protein turnover and degradation that are known to be dysregulated with aging^23^.

In conclusion, physiologic dose of medin induced profound overexpression of pro-inflammatory related genes in human brain microvascular endothelial cells and reduced expression of cell cycle signaling pathway related genes. Medin-induced overexpression of pro-inflammatory genes was reversed by co-treatment with NFκB inhibitor RO106-9920 and monosialoganglioside-containing NLs but was not exacerbated by co-treatment with Aβ42. Medin is a novel therapeutic target to prevent or reverse vascular aging phenotype and NLs represent a promising agent against medin vasculopathy.

## Materials and methods

### HBMVEC treatments

Recombinant medin (> 95% purity) was used for the study and it was obtained following expression in Lemo 21 (DE3) cells using pOPINS-medin; the preparation, purification and characterization methods were described in prior work^8^. Endotoxin levels of < 0.5 ng/mL were confirmed using *Limulus* Amebocyte Lysate assay (Pierce, Dallas TX). Medin was > 95% pure as assessed by sodium dodecyl sulfate–polyacrylamide gel electrophoresis. As peptide control, scrambled medin (SVYLQDQWNNQVQLSRDEVALGIKAKRFGQTNGWSGAGFNTSIDGVAGFV) that was predicted to lack any amyloidogenic hot spots using Aggrescan^24^ was custom-synthesized by Genscript (Piscataway NJ). NL was prepared using phosphatidylcholine, cholesterol and monosialoganglioside (Avanti Polar Lipids, Alabaster AL) at a 70:25:5 molar ratios as previously described using lipid film hydration method^9^. Aβ(1-42) was obtained from Anaspec, (Fremont CA). RO106-9920 was obtained from Tocris Biosciences (Bristol, United Kingdom). Medin and scrambled medin peptide were dissolved in phosphate buffered saline (PBS). Aβ(1-42) was dissolved in 20 µL dimethyl sulfoxide (DMSO) and vortexed then added PBS to prepare 1 mg/mL solution. Medin, scrambled medin and Aβ(1-42) were added to the cell cultures in non-aggregated/soluble forms.

Primary HBMVECs (Cell Systems, Kirkland WA, passages 6–8) were used for the experiments. For RNA-seq experiments (N = 3 biologic replicates per treatment group), HBMVECs were treated for 20 h with medin (5 µM, a dose within physiologic range found in human vascular tissue of 0–13.7 µM^8^), Aβ(1-42) (2 µM, biologically relevant dose that induced endothelial dysfunction in human tissue^13^), NL (300 µg/mL), medin + Aβ(1-42), medin + NL, or no treatment control. For rt-PCR experiments, HBMVECs were treated for 20 h with no treatment control, medin 5 µM without or with RO106-9920 (10 µM) or RO106-9920 (10 µM) (N = 5 biologic replicates per treatment). HBMVECs were treated for 20 h with no treatment control, medin 5 µM without or with NL (300 µg/mL) or NL (N = 5 biologic replicates per treatment). For viability experiments, HBMVECs were treated for 20 h with vehicle, medin 5 µM, medin 5 µM with NL (300 µg/mL), NL (300 µg/mL), medin 5 µM with RO106-9920 (1 or 10 µM) or RO106-9920 (1 or 10 µM) (N = 5 biologic replicates per treatment).

### RNA-seq methods

Seven treatments, each with three replicates, were sequenced using paired-end sequencing, generating two FASTQ files per sample. The raw sequencing data was processed using *Cutadapt* (v4.3)^25^ to remove adapter sequences, and the quality of the processed data was checked with *FASTQC* (v0.12.1, http://www.bioinformatics.bbsrc.ac.uk/projects/fastqc/). Processed reads were then mapped to the human reference genome (GRCh38.p13) using *STAR* (v2.7.10b)^26^ with the Ensembl 106 annotation file to obtain the gene counts for each sample. Gene counts were normalized to library size using the trimmed mean of M-values (TMM) method. One outlier sample was discarded based on the PCA and heat map. Differential gene expression analysis was performed using *edgeR* (v3.40.0)^27^.

### RT-PCR

After cell lysis, RNA was extracted and converted to cDNA using Aurum Total RNA Mini Kit and using iScript cDNA synthesis kit (Bio-Rad Laboratories, Coralville IA) similar to previous methods^4^. β-actin served as reference normalization gene. The following primers were used (Integrated DNA Technologies, Inc., Coralville, IA): IL-8 (Exon Location: 1-1), ICAM-1 (Exon Location: 2- 3) and VCAM-1 (Exon Location: 2a-3) and β-Actin (F: 5′-GAC AGG ATG CAG AAG GAG ATT-3′; R:5′-TGA TCC ACA TCT GCT GGA AGG -3′).

### Viability

Viability was assessed using a commercially available kit (Live/Dead Cytotoxicity Kit, Invitrogen, Carlsbad, CA). Cells were plated onto glass coverslips coated with Attachment Factor. After allowing them to attach for 24 h, followed by exposure to treatment conditions. Cultures were then incubated in DPBS containing ethidium homodimer (membrane impermeable and will only bind to DNA in dead cells) and calcein-AM (converted by esterases found within living cells) for 30 min. The glass coverslips were mounted on slides and imaged using Keyence Fluorescence Microscope BZ-X810 (Itasca, IL) (Excitation/Emission 545/605 for ethidium and 470/525 for calcein-AM). The percentage of dead cells was determined as the proportion of dead cells to the total number of live + dead cells.

### Statistical analyses

For RNAseq, genes that had a count per million (CPM) of greater than 1 in fewer than two samples across the 21 samples were filtered out. The remaining genes were analyzed using a generalized linear model (GLM) with a quasi-likelihood F-test^27^. Additionally, to correct for any batch effects, batch was included in the model. Gene set enrichment analysis (GSEA) was performed using *clusterProfiler* (v4.7.1)^28^.

For RT-PCR and viability experiments involving medin and RO106-9920 or NL co-treatments, one-way repeated measures analysis of variance (ANOVA) with post-hoc pairwise analyses using Holm-Sidak was used (Sigmastat 3.5, Systat Software) using raw data that are normally distributed and have equal variance. For data that do not meet these criteria, natural log transformation was first performed and the transformed data used for ANOVA after meeting criteria for normality and equal variance.

### Supplementary Information

Supplementary Table 1.

Supplementary Table 2.

Supplementary Table 3.

Supplementary Information 4.

## Data Availability

The datasets generated and/or analyzed during the current study are available in Gene Expression Omnibus (GEO) Accession Number GSE235915 (GEO Accession viewer (nih.gov)).
