# Supplementary material for: Transcriptomic analyses reveal proinflammatory activation of human brain microvascular endothelial cells by aging-associated peptide medin and reversal by nanoliposomes
Source: Sci Rep. 2023 Nov 1;13:18802. doi: 10.1038/s41598-023-45959-7 (PMC10620412; doi:10.1038/s41598-023-45959-7)

**Supplement Tables Legend**

**Supplement Table 1. Gene expression profiles.** The table shows gene expression levels in each sample as the log2-transformeed trimmed mean of M-values (TMM) values after filtering out low expressing genes (the count per million of greater than 1 in fewer than two samples across the 21 samples).

**Supplement Table 2. Differential expression over the Vehicle control samples.** The table shows the log2 fold change of expression level for each treatment group over the Vehicle control and the corresponding *P*-values and false discovery rates (FDR).

**Supplement Table 3. Enrichment of KEGG pathways in differentially expressed genes.** The table lists pathway enrichment statistics in differentially expressed genes for each treatment group over Vehicle. Set Size refers to the number of genes in each pathway. Enrichment Score (ES) was calculated by Gene Set Enrichment Analysis (GSEA), which were normalized for the gene set size in each pathway to Normalized Enrichment Score (NES).

**Supplement Figure 1 Legend**

**Supplement Figure 1. Global transcriptomics profiles of medin-, Aβ-, and NL-treated HBMVECs before removing an outlier in the scrMedin group.** A. PCA was applied to gene expression data on 7 different treatment groups, each in triplicates. The x-axis represents the first principal component, which accounts for 23% of total variance in the data, while the y-axis corresponds to the second principal component, explaining 17% of the variance. An outlier in the scrMedin group (indicated by an arrow) was excluded from further analyses. Samples are colored based on their respective treatment. B. The heat map with all samples (including the outlier, indicated by an arrow) shows row- or gene-wise Z score transformed gene expression levels of 7704 DEGs identified by pairwise linear model analysis. Each row represents a gene, while each column corresponds to an individual sample. The columns are sorted by treatments, while the rows are clustered based on the Euclidean distances.

**Supplement Figure 1.**


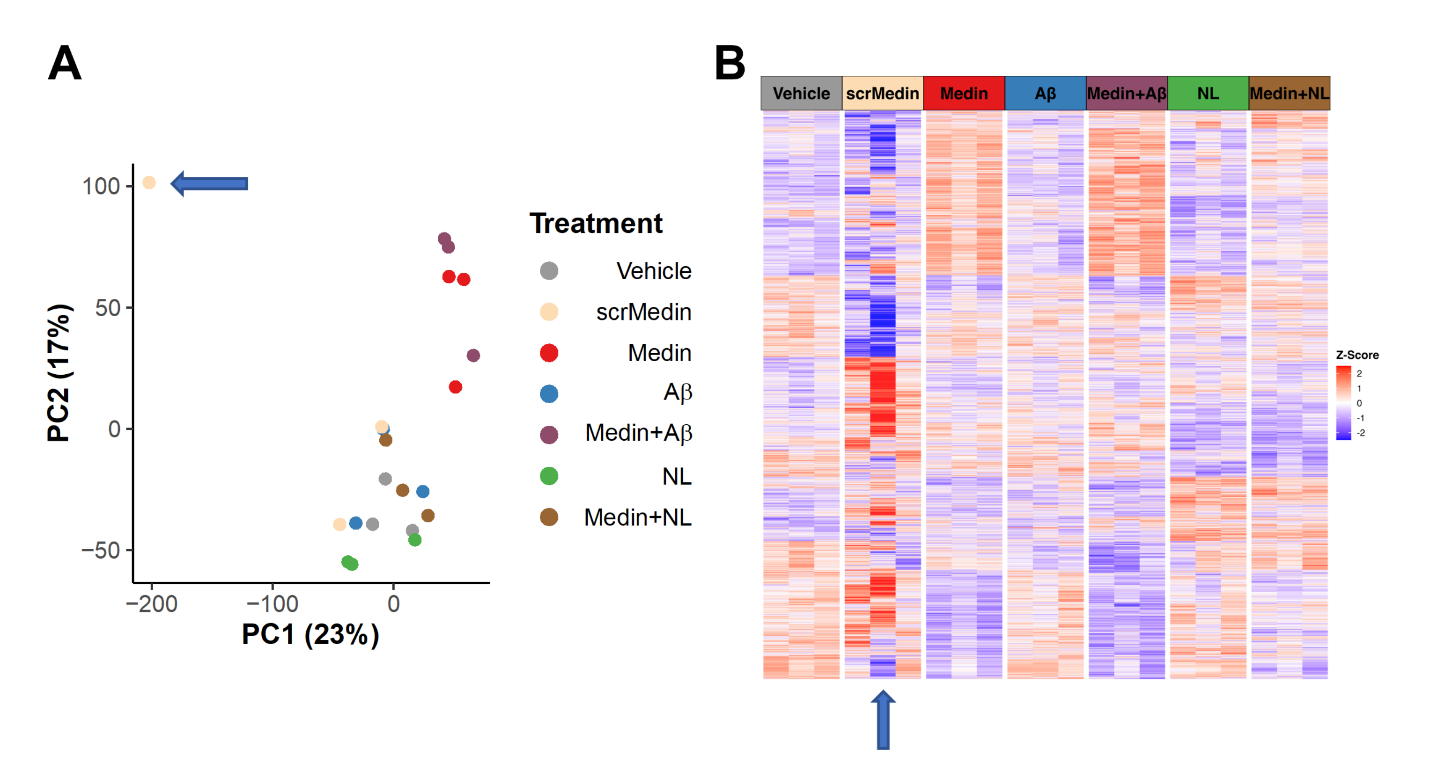

Supplement: Supplementary file 4 — Supplementary Information 4. [file 41598_2023_45959_MOESM4_ESM.docx]
